# Physical quality characteristics of breast and leg meat of slow- and fast-growing broilers raised in different housing systems

**DOI:** 10.5194/aab-63-337-2020

**Published:** 2020-09-14

**Authors:** Melahat Özbek, Metin Petek, Sena Ardıçlı

**Affiliations:** 1 Department of Animal Science, Faculty of Veterinary Medicine, Bursa Uludag University, Bursa, Turkey; 2 Department of Genetics, Faculty of Veterinary Medicine, Bursa Uludag University, Bursa, Turkey

## Abstract

This study was made to determine the effects of genotype and housing system on physical quality characteristics of breast and leg meat of broilers under experimental conditions. The 150 slow-growing and 150 fast-growing 1 d old chicks were divided into three sub-groups with *indoor raised slatted plastic floor*, *indoor concrete floor with rice hull litter*, and *free-range housing* systems (2 genotype groups × 3 housing systems). All birds were offered the same diet and were housed in similar conditions until they were 56 d old. At slaughter, 10 birds from each main group were selected randomly to determine the quality characteristics of the meat. In total, 60 breast meat pieces (pectoralis major muscle) and 60 legs of the chickens were used for meat quality analysis including pH, shear force, and colour characteristics such as lightness ($L^{*}$), redness ($a^{*}$), yellowness ($b^{*}$), saturation ($C^{*}$), and hue angle ($h^{*}$). The pH of breast meat was significantly affected by genotype and housing system (P<0.001 and P<0.001). There were significant genotype × housing system interactions for pH (P<0.015 and P<0.001) and shear force values (P<0.007 and P<0.012) of leg and breast meat. There were no significant effects of genotype and housing system on leg and breast meat colour properties except for effects of genotype on redness ($a^{*}$) of breast meat (p<0.005) and effects of housing on redness of leg meat colour (p<0.031). Slow-growing chickens and chickens housed in deep litter had a higher redness (darker) value of breast and leg meat colour compared to fast-growing birds and free range and slatted floor. In conclusion, it can be said that fast-growing broilers may be more appropriate for slatted plastic floor housing and slow-growing broilers may be more suitable for a free-range housing system, but further research on factors affecting meat quality would be very beneficial, especially in slow-growing broilers.

## 1 Introduction

There is a new trend in poultry meat consumption with a strong demand for meat from production systems, which ensures food security and animal welfare combined with environmental responsibility, consumer health, and better quality of the product. As a result of these, different housing and husbandry systems are becoming widespread in broiler meat production besides conventional production (Stadig et al., 2016; Mir et al., 2017; Sanchez-Casanova et al., 2020). The free-range broiler is one of the most promising broiler meat production systems, where production takes place with slow-growing strains and the meat presents different sensory characteristics (Devatkal et al., 2019). There is a limited number of scientific findings that emphasize qualitative features of free-range production or alternative broiler meat production systems compared to the conventional system. Most authors reported a lower final body weight, poorer feed conversion efficiency, and better meat quality in free-range housing systems compared to conventional rearing (Bogosavljevic-Boslovic et al., 2012). Slow-growing genotypes are becoming popular in free-range production and organic chicken meat production due to some welfare problems of fast-growing broilers in conventional production (Hartcher and Lum, 2019; Louton, 2019).

The colour of broiler meat, both cooked and raw, is the best indicator for wholesomeness, and it is one of the main factors affecting a customer's preference at the point of sale (Font-i-Furnols and Guerrero, 2014). Identification of colour is an easy way to determine the meat pH. If the meat is very dark, it will have a high pH; if it is light, it will have a low pH (Anadon, 2002). Several factors affecting poultry meat colour (including genetics, sex, age, feed, pre-slaughter handling, and slaughter) have been reported (Krallik et al., 2018). There are some differences in the quality of pigmentation in broiler chickens, and the level of pigmentation is hereditary (Zhuang and Savage, 2013). Bianchi et al. (2006) found no differences in broiler breast meat colour based on the bird genotype. When comparing five commercial broiler strains, Mehaffey et al. (2006) found no significant differences in breast meat brightness among the birds. Similar to this finding, Brewer et al. (2012) found that the strain of broiler did not have a significant effect on breast fillet colour. However, Abdullah et al. (2010) found significant differences in the breast meat colour values between two broiler genotypes. Comparing a slow-growing French label-type line and a fast-growing standard broiler genotype of commercial chickens, Debut et al. (2003) found that the breast and leg meat of the fast-growing line was lighter than that of the breast and leg meat of the slow-growing line. Da Silva et al. (2017) showed that the free-range broiler meat (slow-growing) had higher yellow colour and shear force and lower red colour and pH compared to the industrial broiler meat (fast-growing) due to intensive physical activity of the birds and pre-slaughter stress. The aim of the study was to compare physical quality characteristics of breast and leg meat of slow- and fast-growing broilers housed in a barn (indoor deep litter and indoor slatted floor) and free-range housing systems.

## 2 Materials and methods

This study examined the effects of different housing systems for free-range (including indoor and outdoor ranges), indoor deep-litter, and indoor raised slatted plastic floor systems; we also examined the effects of two genotypes of broilers (slow- and fast-growing broilers) on breast and leg meat quality. There were six main groups (2 genotype × 3 housing systems) and five replicates of each main group in the study. The indoor part of the free-range and deep-litter housing systems consisted of a concrete floor with rice hull litter. The experimental protocol was approved by the Animal Policy and Welfare Commission of Bursa Uludag University in Turkey.

### 2.1 Management

In total, 150 slow-growing and 150 fast-growing 1 d old chicks were raised in the experimental unit of the faculty farm for this experiment. Each main group consisted of 50 male chicks with five replicates including 10 birds in each replicate. A continuous light regime consisting of daylight and artificial light was used in the first 7 d of the experiment. Daylight and an intermittent lighting programme consisting of 2 h of dark and 2 h of light during the night were applied from the 8th day until the end of the experiment. All birds were fed with a commercial multiphase diet (starter from days 0 to 10, grower I from days 11 to 23, grower II from days 24 to 36, and finisher from days 37 to 56), which was produced by a commercial feed company in Turkey.

At slaughter, 10 birds from each main group were selected randomly to determine the quality characteristics of the meat. Feed was withdrawn approximately 12 h before slaughter. After the slaughter in standard conditions, the carcasses were scalded in a 55 ${}^{\circ }$C water bath for 20 s and defeathered in a rotary drum picker (Nielsen et al., 2019). After whole carcasses were chilled for 3 h at 4 ${}^{\circ }$C, the breast and legs were removed from each carcass. In total, 60 breast meat pieces and 60 legs of the chickens were used to perform the comparison of pH, shear force, and colour characteristics.

### 2.2 Data

The carcass weights were determined first, and carcass parts as whole breasts and two legs including skin and bone were removed from the whole carcasses. All cut-up pieces were weighed, and their yields were calculated. The pH analysis was carried out with a penetration electrode at three different points of the chicken muscle using a calibrated pH meter (Hanna HI99163). Shear force (SF) of meat was evaluated using cooked samples. After cooking, the samples were cooled to room temperature and stored at 4 ${}^{\circ }$C for 24 h. Approximately 3–5 cm${}^{3}$ of the sample taken from the middle of the legs and breast meat was weighed before and after cooking. The texture was measured using the texture analyser (Zwick/Roell 20.05, Germany) using the Warner-Bratzler cutting blade head to measure the shear force (Zhuang and Savage, 2013).

Colour determinations of the meat samples were performed with a colorimeter (Konika Minolta Chroma Meter CM 600d, Minolta GmbH, Langenhagen, Germany) programmed with a CIE $L^{*}a^{*}b^{*}$ system (D65 illuminant, 10${}^{\circ }$). The analysis was carried out on the medial surface (bone side) of the left breast and left leg muscle at 24 h post-mortem (Keskin et al., 2017). The colorimeter was calibrated using the specific whiteboard before measurement began. Each value was an average of three measurements from an area of the meat between 4–5 cm${}^{2}$ to get a representative evaluation of the samples. The $L^{*}$ value is the lightness component, which ranges from 0 to 100 (from black to white); $a^{*}$ and $b^{*}$ both range from -60 to +60 with $a^{*}$ ranging from green if negative to red if positive and $b^{*}$ ranging from blue if negative to yellow if positive (Kralik et al., 2018). Subsequently, the colour intensity/saturation index (chroma value, $C^{*}=(a^{*2}+b^{*2}{)}^{0.5}$) and hue angle as arctangent (arctan) values $\left(h^{*}={\mathrm{tan}}^{-1}(b^{*}/a^{*}).180/\pi \right)$ were calculated from the $a^{*}$ and $b^{*}$ values for each meat sample at three sample points to get a representative evaluation of the samples (Ingram et al., 2008).

### 2.3 Statistical analysis

All statistical analyses for the traits were performed using SPSS^®^ computer software 13.00 (IBM SPSS 2011). Analysis of variance was used to analyse the effects of (and interactions between) housing systems and genotype of broilers (Snedecor and Cochran, 1989). The general form of the model used in the analyses was the following:

Yijk =μ+Ai+Bj+A×B+eijk,

where A represents the effects of housing system, and B represents the effects of genotype; A × B represents an interaction. Also, i can have values of 1, 2, or 3 (where 1 is for deep litter, 2 is for slats, and 3 is for free-range housing), and j can have values of 1 or 2 (where 1 is for fast-growing genotype and 2 is for slow-growing genotype). μ is a constant and e is an error term.

**Table 1:** Average live weight, carcass weight, and leg and breast weight; pH; and shear force values of meat samples of slow- and fast-growing broilers from different housing systems.

Groups	Live	Carcass	Leg weight	Breast	pH		Shear force	
	weight	weight		weight			(max force) (N)	
	g	g	g	g	Leg	Breast	Leg	Breast
Genotype								
Fast (F)	4403 ± 79	3589 ± 63	1507 ± 21	1586 ± 33	6.10 ± 0.03	5.73 ± 0.019	17.12 ± 1.81	33.07 ± 2.91
Slow (S)	2386 ± 77	1826 ± 62	832 ± 20	647 ± 32	6.12 ± 0.04	5.56 ± 0.018	18.35 ± 1.80	21.88 ± 2.90
Housing system								
Free-range (FR)	3643 ± 95${}^{\text{a}}$	2879 ± 75${}^{\text{a}}$	1224 ± 25${}^{\text{a}}$	1232 ± 39${}^{\text{a}}$	6.16 ± 0.04	5.71 ± 0.021${}^{\text{a}}$	20.30 ± 2.21	24.97 ± 3.56
Slat (SLAT)	3234 ± 97${}^{\text{b}}$	2572 ± 77${}^{\text{b}}$	1128 ± 25${}^{\text{b}}$	1045 ± 40${}^{\text{b}}$	6,07 ± 0.05	5.69 ± 0.023${}^{\text{a}}$	15.18 ± 2.22	31.37 ± 3.55
Deep Litter (DL)	3307 ± 95${}^{\text{b}}$	2671 ± 75${}^{\text{b}}$	1157 ± 25${}^{\text{b}}$	1073 ± 39${}^{\text{b}}$	6.09 ± 0.04	5.54 ± 0.022${}^{\text{b}}$	17.74 ± 2.21	26.09 ± 3.56
Genotype × housing system								
F × FR	4817 ± 130${}^{\text{A}}$	3908 ± 107${}^{\text{A}}$	1612 ± 35${}^{\text{A}}$	1790 ± 55${}^{\text{A}}$	6.15 ± 0.06${}^{\text{A}}$	5.80 ± 0.033${}^{\text{A}}$	13.40 ± 3.14${}^{\text{B}}$	21.22 ± 5.04${}^{\text{B}}$
S × FR	2469 ± 124${}^{\text{a}}$	1849 ± 106${}^{\text{a}}$	837 ± 34${}^{\text{a}}$	674 ± 55${}^{\text{a}}$	6.18 ± 0.05${}^{\text{a}}$	5.62 ± 0.032${}^{\text{a}}$	27.20 ± 3.13${}^{\text{a}}$	28.72 ± 5.03${}^{\text{a}}$
F × SLAT	4116 ± 141${}^{\text{B}}$	3324 ± 112${}^{\text{B}}$	1418 ± 37${}^{\text{B}}$	1456 ± 58${}^{\text{B}}$	6,17 ± 0.06${}^{\text{A}}$	5,83 ± 0.033${}^{\text{A}}$	16,81 ± 3,13${}^{\text{B}}$	42,94 ± 5,03${}^{\text{A}}$
S × SLAT	2352 ± 134${}^{\text{a}}$	1820 ± 106${}^{\text{a}}$	838 ± 35${}^{\text{a}}$	633 ± 55${}^{\text{a}}$	5.97 ± 0.05${}^{\text{b}}$	5.54 ± 0.031${}^{\text{b}}$	13.54 ± 3.14${}^{\text{a}}$	19.80 ± 5.04${}^{\text{b}}$
F × DL	4277 ± 114${}^{\text{B}}$	3534 ± 107${}^{\text{B}}$	1492 ± 35${}^{\text{B}}$	1513 ± 55${}^{\text{B}}$	5.98 ± 0.05${}^{\text{B}}$	5.57 ± 0.032${}^{\text{B}}$	21.16 ± 3.13${}^{\text{A}}$	35.06 ± 5.03${}^{\text{A}}$
S × DL	2335 ± 133${}^{\text{a}}$	1807 ± 106${}^{\text{a}}$	822 ± 36${}^{\text{a}}$	632 ± 55${}^{\text{a}}$	6,20 ± 0.06${}^{\text{a}}$	5.50 ± 0.033${}^{\text{b}}$	14.32 ± 3.14${}^{\text{a}}$	17.12 ± 5.02${}^{\text{b}}$
Level of significance								
Genotype (G)	0.001	0.001	0.001	0.001	0.741	0.001	0.636	0.012
Housing (H)	0.008	0.020	0.026	0.003	0.340	0.001	0.283	0.411
G × H	0.095	0.043	0.029	0.028	0.015	0.009	0.007	0.012

a–b and A–B represent means ± SEM (standard error of the mean) with different superscripts that vary significantly within the row.

**Table 2:** Colour values of breast meat collected from slow- and fast-growing broilers raised in three different housing systems (mean ± SEM).

Groups	Lightness, $L^{*}$	Redness, $a^{*}$	Yellowness, $b^{*}$	Chroma, $C^{*}$	Hue, $h^{\text{o}}$
Genotype					
Fast growing	55.54 ± 1.3	-0.93 ± 0.2	8.48 ± 0.4	8.61 ± 0.5	-0.82 ± 0.4
Slow growing	58.05 ± 1.2	0.04 ± 0.2	9.38 ± 0.5	9.41 ± 0.5	-0.11 ± 0.3
Housing system					
Free-range	56.72 ± 1.5	0.05 ± 0.3	9.58 ± 0.6	9.60 ± 0.5	-0.00 ± 0.5
Slats	56.12 ± 1.6	-0.80 ± 0.2	8.31 ± 0.5	8.42 ± 0.6	-0.84 ± 0.6
Deep litter	57.55 ± 1.4	-0.59 ± 0.3	8.91 ± 0.6	9.01 ± 0.4	-0.55 ± 0.5
Genotype × housing system					
Fast × free-range	56.23 ± 2.3	-0.04 ± 0.4	9.74 ± 0.8	9.66 ± 0.8	-0.31 ± 0.5
Slow × free-range	57.21 ± 2.3	0.15 ± 0.4	9.42 ± 0.6	9.44 ± 0.7	-0.30 ± 0.6
Fast × slats	53.35 ± 1.9	-1.57 ± 0.3	7.98 ± 0.8	8.17 ± 0.8	-1.36 ± 0.7
Slow × slats	58.88 ± 2.2	-0.03 ± 0.4	8.64 ± 0.7	8.67 ± 0.6	-0.32 ± 0.4
Fast × deep litter	57.03 ± 2.3	-1.18 ± 0.3	7.73 ± 0.8	7.90 ± 0.8	-0.78 ± 0.6
Slow × deep litter	58.10 ± 2.1	-0.00 ± 0.4	10.09 ± 0.9	10.12 ± 0.8	-0.32 ± 0.6
Level of significance					
Genotype	0.189	0.005	0.193	0.239	0.193
Housing	0.820	0.088	0.322	0.362	0.436
Genotype × housing system	0.528	0.212	0.276	0.297	0.898

## 3 Results

The results of the average carcass, leg, and breast weights; pH; and shear force values of meat samples are displayed in Table 1. In this study, the whole-carcass, breast, and leg meat weights were significantly influenced by genotype and housing system. There were significant differences for breast meat shear force value between the slow- and fast-growing genotypes (P<0.012). Genotype and housing system were found to have a significant effect on breast meat pH (P<0.001). The genotype × housing system interaction was significant for all carcass characteristics, meat pH, and shear force traits.

The colour properties of breast meat collected from slow- and fast-growing broilers raised in three different housing systems are presented in Table 2. Concerning the effect of the housing system on broiler breast meat colour, no differences were found for colour parameters ($L^{*},a^{*},b^{*},C^{*}$, and $h^{*}$). Fast-growing broilers exhibited a significantly lower redness value compared with slow-growing birds (P<0.005).

**Table 3:** Colour values of leg muscles of slow- and fast-growing broiler chickens either raised with free-range or indoor systems (either fully slatted or fully deep litter).

Groups	Lightness, $L^{*}$	Redness, $a^{*}$	Yellowness, $b^{*}$	Chroma, $C^{*}$	Hue, $h^{\text{o}}$
Genotype					
Fast growing	59.31 ± 1.5	2.89 ± 0.5	12.63 ± 0.60	13.04 ± 0.6	0.93 ± 0.2
Slow growing	61.16 ± 1.4	3.84 ± 0.4	13.49 ± 0.59	14.17 ± 0.5	0.90 ± 0.3
Housing system					
Free-range	59.96 ± 1.8	2.20 ± 0.6${}^{\text{b}}$	13.39 ± 0.6	13.68 ± 0.6	0.48 ± 0.3
Slats	60.19 ± 1.9	3.32 ± 0.7${}^{\text{b}}$	12.22 ± 0.7	12.74 ± 0.8	1.01 ± 0.2
Deep litter	60.55 ± 1.9	4.57 ± 0.5${}^{\text{a}}$	13.56 ± 0.7	14.41 ± 0.7	1.26 ± 0.3
Genotype × housing system					
Fast × free-range	60.14 ± 2.6	1.91 ± 0.8	13.33 ± 1.0	13.56 ± 0.9	0.19 ± 0.4
Slow × free-range	59.78 ± 2.5	2.49 ± 0.7	13.45 ± 0.9	13.79 ± 1.1	0.77 ± 0.5
Fast × slats	56.06 ± 2.5	3.66 ± 0.7	11.91 ± 1.1	12.47 ± 1.0	1.27 ± 0.5
Slow × slats	64.32 ± 2.7	2.98 ± 0.9	12.52 ± 1.0	13.00 ± 1.0	0.74 ± 0.4
Fast × deep litter	61.74 ± 2.6	3.11 ± 0.8	12.64 ± 1.1	13.09 ± 1.1	1.34 ± 0.3
Slow × deep litter	59.37 ± 2.7	6.04 ± 0.7	14.50 ± 1.0	15.73 ± 1.0	1.18 ± 0.4
Level of significance					
Genotype	0.407	0.180	0.310	0.221	0.922
Housing	0.975	0.031	0.369	0.332	0.220
Genotype × housing system	0.130	0.115	0.684	0.502	0.453

a–b are means ± SEM with different superscripts that vary significantly within the row.

The effect of genotype and housing system on broiler leg meat colour is shown in Table 3. The birds kept in the deep-litter housing system had a significantly higher redness colour value compared to birds housed in free-range and slatted floor systems (P<0.031). There was no significant effect of genotype on colour values of leg muscles.

## 4 Discussion

The quality of poultry meat is determined by several multi-factorial complex factors (Mir et al., 2017; Zhao et al., 2018). In this study, the effects of genotype and housing system on whole-carcass, breast, and leg meat weights of broilers were found to be significant. As expected, fast-growing broilers had higher carcass and cut-up weights for breast and thighs compared to slow-growing broilers (Devatkal et al., 2019). Interestingly, free-range broilers had significantly heavier carcass, breast, and leg meat weights compared to meat from fully slatted or fully littered flooring. It was found that there was a significant genotype × housing system interaction for carcass, breast, and leg meat weights of the birds (P<0.043, P<0.029, and P<0.028). This revealed that fast-growing free-range broilers had a significantly heavier carcass, breast, and leg weights compared to broilers housed in fully slatted or deep-litter housing, while no significant differences were observed for these parameters for slow-growing birds raised in all three different housing systems.

Many factors affect broiler meat pH and meat colour, such as pre-slaughter management, sex, and diet (Mir et al., 2017). In this study, pH of breast meat was significantly affected by genotype and housing system (P<0.001, P<0.001), whereas results showed that genotype and housing system did not influenced the pH of leg meat (P<0.741 and P<0.34). In general, breast meat pH was found to be higher than the leg meat pH. Mean pH values were between 5.50 and 5.83 for leg meat and between 5.97 and 6.20 for breast meat for the broilers. Contrary to our results, Da Silva et al. (2017) and Cygan-Szczegielniak et al. (2019) reported that the free-range broiler meat had a higher shear force and lower pH in comparison to the commercial broiler meat, due to intensive physical activity in growing phase. The degree of protein denaturation and physical appearance of meat, dependent on post-mortem pH and muscles at pH ≥ 6.0, are characterized by minimal protein denaturation (Anadon, 2002). A significant genotype × housing system interaction for meat pH and shear force revealed that fast-growing broilers raised only in slats showed significantly greater values than slow-growing birds (P<0.009 and P<0.012). Although leg meat pH and shear force traits were not significantly affected by genotype and housing system, genotype × housing system interactions for these traits revealed that slow-growing birds in a free-range housing system and fast-growing birds in fully slatted flooring had significantly higher values. Meat pH has a direct relation to the meat quality attributes such as tenderness, water-holding capacity, colour, juiciness, and shelf life (Mir et al., 2017). The broiler breast meat with high pH has a higher water-binding capacity than meat with lower pH. A direct correlation between the colour of the breast fillets and the pH of the meat has been reported (Fletcher, 1995), and meat pH can be identified by meat colour easily. If the meat is dark, it will have a high pH; if it is light, it will have a low pH (Anadon, 2002).

Meat tenderness is one of the most important quality factors for meat texture, and any variations in tenderness will influence a consumer's decision for poultry meat. The texture and degree of firmness of the meat are a function of the amount of water held intramuscularly. We have evaluated the meat tenderness by shearing with Warner-Bratzler shear blades. In this study, there were no significant differences for the shear force value of leg meat between the two genotypes, but it was significantly greater for breast meat in fast-growing broilers (P<0.012). There was a significant genotype × housing system interaction for leg and breast shear force values. Breast meat shear force values of slow- and fast-growing broilers in free-range housing were found to be significantly higher compared to birds housed in the other two housing groups. During the last few years, consumer interest in slow-growing broiler meat has been steadily increasing. Hence, new strains of slow-growing broilers are being introduced to meet the demand. Devatkal et al. (2019) showed that the shear force value of thigh meat was higher in a new slow-growing broiler line than the commercial fast-growing broiler. Genetic variation among broiler birds could contribute to large differences in meat quality. Heritability estimates for meat quality traits in broilers are amazingly high (0.35–0.81), making genetic selection the best tool for improvement of broiler meat quality (Mir et al., 2017).

The colour of broiler meat is probably one of the most essential visual factors affecting the purchasing decisions of consumers in the market (Wideman et al., 2016). It depends on a large number of factors such as genotype, slaughter age, sex, housing condition, diet, pre-slaughter stress, and some muscle myopathies as white striping (Siekman et al., 2018; Albrecht et al., 2019; Qamar et al., 2019). Almasi et al. (2015) reported that indoor treatment had a lower impact on breast meat than the outdoor system. In a study, it was found that the free-range broiler meat had higher yellow colour ($b^{*}$) and lower red colour ($a^{*}$) values than conventional broiler meat due to intensive physical activity in the growing period (Da Silva et al., 2017). Genotypic differences were more pronounced in the thigh than in breast muscle in broiler meat production. The heritability estimates of lightness (0.50–0.75), redness (0.57–0.81), and yellowness (0.55–0.64) suggest that genetic selection is the best tool for improvement of broiler meat quality (Mir et al., 2017). Berri et al. (2001) reported that selection for higher body weight and breast meat yield led to lighter breast meat in commercial and experimental groups. Devatkal et al. (2019) reported that in fresh and chilled breast meat lightness and redness were not significantly different for the slow-growing and commercial fast-growing groups, and redness and lightness values of the two genotypes did not show significant variation in fresh thigh meat. Consumers connect the meat colour with its freshness, and it can be determined visually or by using instruments. The instrumental determination of meat colour is a more efficient method. Three basic properties can generally characterize meat colour: hue, brightness, and saturation (Salakova, 2012). In our study, we used instrumental determination of the broiler meat colour, and significant differences were observed in the breast muscle colour of slow- and fast-growing chickens regarding the redness ($a^{*}$) of meat samples (P<0.005). As reported previously, slow-growing broilers had a bit more redness (darker) value for their breast meat, probably due to melanin, which has a substantial impact on their meat colour (Muth and Zarate, 2017). Hoan ve Khoa (2016) reported that differences for the lightness ($L^{*}$, 54.15, and 58.12), redness, ($a^{*}$, 2.77, and 1.26), and yellowness ($b^{*}$, 17.73 and 15.43) values of breast meat of a slow- and a fast-growing genotype were found to be significantly important at 49 d of slaughter age. Stadig et al. (2016) found that broiler meat colour characteristics ranged between 53.9 and 55.3 for lightness, between 5.7 and 6.3 for redness, and between 13.4 and 14.7 for yellowness. In a study (Dogan et al., 2019), the $L^{*}$ and $a^{*}$ values of breast meat were found to be higher in slow-growing broilers. On the other hand, Kralik et al. (2014) reported that the chicken genotype did not influence the lightness or yellowness values of chicken meat.

Along with the genotype, some environmental factors can have important effects on broiler meat colour. Bianchi et al. (2006) showed that genotype, live weight, and transportation might influence breast meat colour in broiler chickens. Faria et al. (2009) reported that the ingestion of a larger amount of forage rich in carotenoids by slow-growing chickens provides a higher intensity of yellow colour in the meat, resulting in higher $b^{*}$ values. Using a wheat-based diet tends to lighten the colour of breast meat but has less effect on thigh meat. Slaughter age was also observed to have an affect on the redness of breast meat (Coban et al., 2014). There was a negative relationship between meat colour characteristics with brightness ($L^{*}$), redness ($a^{*}$), and cooking loss in broiler chickens (Janisch et al., 2011). Da Silva et al. (2017) observed an inverse correlation between lightness, pH, and shear force values of broiler meat.

The main issues with broiler meat colour are muscle type; raw breast meat exhibits a pale pink colour, while the raw thigh and leg meat appear dark red (Mir et al., 2017). In our study, if we compared breast and leg meat, leg meat of the birds was found darker than breast meat in both genotypes. It is well documented that the darker colour of leg meat is due to the higher concentrations of myoglobin and haem pigments, as well as a higher pH when compared to breast meat (Wideman et al., 2016). In this study, there were no significant effects of the housing system on all breast meat colour properties. Similarly, Połtowicz and Doktor (2011) reported that there were no significant differences for $L^{*}$, $a^{*}$, and $b^{*}$ values of free-range broiler meat. Michalczuk et al. (2017) showed no significant differences in the breast meat colour of the slow-growing broiler raised in indoor and outdoor systems. Woo-Ming et al. (2018) found no significant effects of range access on breast meat colour in free-range broilers, whereas Da Silva et al. (2017) showed that the redness ($a^{*}$) scores for the conventional indoor broiler chicken meat were higher than those of the free-range broiler meats. It was found that $L^{*}$ (Stadig et al., 2016) and $b^{*}$ values (Batkowska et al., 2015; Stadig et al., 2016) of free-range breast meat were greater than broiler meat produced conventionally. Połtowicz and Doktor (2011) reported that lightness, redness, and yellowness values of broiler meat were changed between 53.09 and 53.90, between 14.70 and 15.96, and between 6.84 and 7.76, respectively, and the rearing system had no effect on these characteristics. In a previous study, Michalczuk et al. (2017) showed that $L^{*}$, $a^{*}$, and $b^{*}$ values of broiler breast meat and the $a^{*}$ value of thigh muscle did not differ in broilers raised in free-range and conventional indoor systems, while $L^{*}$ and $b^{*}$ values of thigh muscle were lower in broiler chickens grown in the free-range system.

In this study, colour values of leg muscles obtained from slow- and fast-growing broilers were not significantly different. Similar to our results, Doğan et al. (2019) showed that the values of leg meat brightness, redness, and yellowness were higher in the slow-growing broilers, but the differences were not significantly important. On the other hand, Almasi et al. (2015) reported that leg and breast skin and muscle of slow-growing broiler chickens were significantly darker, and breast skin was yellower compared to the medium-growing birds. Northcutt et al. (1998) and Grashorn (2006) reported that there was no significant effect of genotype on brightness and yellowness of meat colour characteristics and that genetic effect is the only significant effect on redness. Similar to these findings, Zhuang and Savage (2013) reported that there were differences in broiler meat pigmentation quality, and the level of pigmentation was hereditary. Cruz et al. (2018) investigated breast and leg meat quality of five different broiler genotypes. They reported that leg meat had a greater brightness, redness, hue angle, and chroma values than breast meat.

In this study, there was a significant effect of the housing system on the redness ($a^{*}$) of leg muscles of broiler meat. The leg redness feature was found to be the darkest in the deep-litter housing system (P<0.031). Especially the leg meat of slow-growing birds raised in the deep-litter housing system had a higher $a^{*}$ value than the others, probably due to deterioration of litter quality with advancing growth period. The floor is in direct contact with the bird throughout the growing period, and high moisture is probably the single most influential factor leading to carcass problems from the litter (Dunlop et et al., 2016). Da Silva et al. (2017) showed that free-range broiler meat had a higher $b^{*}$ and lower $a^{*}$ values compared to the conventional broiler. However, Woo-Ming et al. (2018) reported that access to pasture did not affect breast colour parameters in broiler chickens. Viana et al. (2017) found that the $a^{*}$ value of broiler meat was higher in organic production, and the $b^{*}$ value was higher in conventional production.

## 5 Conclusions

Broiler meat quality is dependent on multiple factors and thus a very complex process. The genotype, housing system, and their interaction did not influence broiler meat colour, except for redness of the meat. The majority of colour parameters of breast and leg muscles were similar in the groups. Taking into account genotype × housing system interactions for meat pH and shear force traits, fast-growing broilers may be more appropriate for fully slatted housing and slow-growing broilers may be more suitable for the free-range housing system. Slow-growing broiler chickens can be an alternative poultry meat for consumers, but further research on the factors affecting meat quality would be beneficial, especially in the slow-growing broilers and those kept on slatted flooring.

## Data Availability

Data are available from the corresponding author upon request.
